# Effect of *Streptomyces* probiotics on the gut microbiota of *Litopenaeus vannamei* challenged with *Vibrio parahaemolyticus*

**DOI:** 10.1002/mbo3.967

**Published:** 2019-11-18

**Authors:** José Manuel Mazón‐Suástegui, Joan Sebastian Salas‐Leiva, Ricardo Medina‐Marrero, Ricardo Medina‐García, Milagro García‐Bernal

**Affiliations:** ^1^ Centro de Investigaciones Biológicas del Noroeste (CIBNOR) La Paz B.C.S México; ^2^ Cátedra‐CONACyT Departamento de Medio Ambiente y Energía Centro de Investigación en Materiales Avanzados Chihuahua México; ^3^ Centro de Bioactivos Químicos Universidad Central de Las Villas Santa Clara Villa Clara Cuba; ^4^ Facultad de Química‐Farmacia Universidad Central de Las Villas Santa Clara Villa Clara Cuba

**Keywords:** *Bacteriovorax*, *Litopenaus vannamei*, Microbiome, *Streptomyces*, *Vibrio*

## Abstract

This study assessed the intestinal microbiota of juveniles of the White shrimp *Litopenaus vannamei*, whose feed was enriched with three probiotic formulations: *Streptomyces* sp. RL8 (RL8); a mix of *Lactobacillus graminis* and *Streptomyces* spp. RL8 and N7 (Lac‐Strep); and a mix of *Bacillus* spp. and *Streptomyces* spp. RL8 and N7 (Bac‐Strep). The analysis was performed by sequencing the V3 region of the 16S rRNA gene of treated animals and the control group before and after *Vibrio parahaemolyticus* challenge. After challenge, the highest Shannon diversity indexes corresponded to RL8 and Bac‐Strep (3.94 ± 0.11 and 3.39 ± 0.3, respectively) and the lowest to the control group (2.58 ± 0.26). The most abundant phyla before and after challenge were *Proteobacteria*, *Actinobacteria,* and *Bacteroidetes*. The principal component analysis and Statistical Analysis of Metagenomic Profiles (STAMP) showed that the gut microbiota of the groups RL8 and Bac‐Strep after challenge was different from the other experimental groups, which was characterized by a higher bacterial diversity, as well as a significant stimulation of the *Bacteriovorax* population and other antimicrobial producing genera that protected shrimp from infection.

## Graphical abstract

We have shown the modulating effect of Streptomyces sp. RL8 alone and combined with Bacillus, but not with Lactobacillus, over the gut microbiota of the white shrimp Litopenaeus vannamei challenged with Vibrio parahaemolyticus. This effect was characterized by a higher bacterial diversity, as well as a significant stimulation of the predatory Bacteriovorax population and other antimicrobial producing genera that protected shrimp from infection.

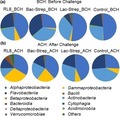

## 1 INTRODUCTION

The Pacific White shrimp *Litopenaeus vannamei* is the main shellfish species reared worldwide. However, it is susceptible to several pathogenic microorganisms that can cause severe economic losses to the aquaculture industry (Tsai et al., 2014; Tzuc, Escalante, Rojas Herrera, Gaxiola Cortes, & Ortiz, 2014). In an aquaculture system, shrimp and microorganisms share the same aquatic medium; thus, the intestinal microbial community interacts directly with planktonic microbiota (De Schryver & Vadstein, 2014; Xiong et al., 2015). Therefore, characterization of the intestinal microbiota (IM) of aquatic organisms is a priority to understand host–microorganism interactions and the corresponding relationship with the surrounding microbiota (Gillilland et al., 2012; Roeselers et al., 2011).

The growing knowledge of the role of the IM to the host health has generated a wide interest to modulate its composition and metabolic function to benefit aquaculture production. Thus, numerous strategies have been developed to improve the colonization of the gastrointestinal tract (GIT) of aquatic animals with beneficial bacteria and avoid proliferation of pathogenic bacteria. One of such approaches has been diets with pre‐, pro‐, and symbiotic supplementation, which can also improve animal growth and feed efficiency (Ringø, Olsen, Jensen, Romero, & Lauzon, 2014). Therefore, probiotics have shown to be a promising and environmentally friendly alternative for disease prevention, especially in crustacean aquaculture of high commercial value (Lobo et al., 2014).

Several studies have indicated that probiotics could contribute to enzymatic digestion, inhibit pathogenic microorganisms, promote growth factors, and increase the immune response of aquatic organisms (Krummenauer et al., 2014). Consequently, new beneficial microorganisms that could be used as probiotics in aquaculture are constantly explored (Lazado, Caipang, & Estante, 2015). Marine actinomycetes are among those promising candidates by virtue of their ability to produce a wide variety of antibiotics and extracellular enzymes (Barka et al., 2016; Prakash et al., 2013). In fact, some studies have shown that marine strains of the genus *Streptomyces* increased growth, survival and resistance to disease in the shrimp *Penaeus monodon* (Augustine, Jacob, & Philip, 2016; Das, Lyla, & Ajmal Khan, 2006; Das, Ward, & Burke, 2010).

Previous experiments have also shown the in vitro probiotic effect of *Streptomyces* spp. isolated from marine sediments of Cuba (García‐Bernal et al., 2015), as well as the increased resistance to infection and survival of *L. vannamei* juveniles treated with those strains and challenged with *V. parahaemolyticus* CAIM 170 (García‐Bernal, Medina‐Marrero, Campa‐Córdova, & Mazón‐Suástegui, 2017). Therefore, the objective of this research was to determine the effect of *Streptomyces* strains on the intestinal bacterial community in juveniles of the White shrimp *L. vannamei*, as part of a previous study revealing the probiotic effect of *Streptomyces* strains alone or combined with *Bacillus* and *Lactobacillus* (García‐Bernal, Medina‐Marrero, et al., 2017).

## 2 MATERIALS AND METHODS

### 2.1 Test organisms

**Figure 1:**
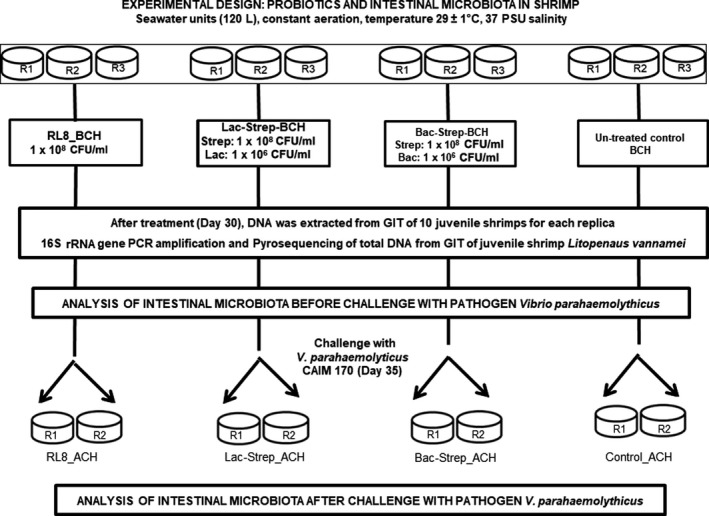
Detailed experimental design to assess the effect of *Streptomyces* alone [RL8] and combined with *Bacillus* [Bac‐Strep] and *Lactobacillus* [Lac‐Strep] on the gut microbiota of *Litopenaeus vannamei*, before [ˍBCH] and after [ˍACH] challenge with *V. parahaemolyticus*

The *Streptomyces* spp. RL8 and N7 isolated from marine sediments of Cuba (García‐Bernal et al., 2015), a *Bacillus* (Bac) mixture composed of *Bacillus tequilensis* (YC5‐2), *B*. *endophyticus* (C2‐2), and *B*. *endophyticus* (YC3‐b) (Luis‐Villaseñor, Macías‐Rodríguez, Gómez‐Gil, Ascencio‐Valle, & Campa‐Córdova, 2011), and the *Lactobacillus graminis* (Lac) strain, with proven probiotic activity from the bacterial collection of Centro de Investigaciones Biológicas del Noroeste (CIBNOR) (Abasolo‐Pacheco et al., 2016), were used as probiotic agents. Four experimental groups with three replicates each were used in the experiment as follows: (a) RL8 (*Streptomyces* sp. RL8); (b) Lac‐Strep (*Lactobacillus graminis* + *Streptomyces* sp. RL8 and *Streptomyces* sp. N7; 1:1:1 proportion); (c) Bac‐Strep (*B. tequilensis* YC5‐2, *B. endophyticus* C2‐2, *B. endophyticus* YC3‐B, *Streptomyces* sp. RL8, and *Streptomyces* sp. N7; 1:1:1:1:1 proportion); and (d) control group (no probiotics added). The treated shrimps were provided a commercial pelletized feed (Purina®, Ciudad Obregón, SON, MX, 35% protein) in which the probiotic suspensions were incorporated by spraying. The *Lactobacillus* and *Bacillus* strains were incorporated at a final concentration of 1 × 10^6^ colony‐forming units (CFU) /g of feed (Abasolo‐Pacheco et al., 2016; Luis‐Villaseñor et al., 2011), whereas *Streptomyces* strains were added at a ratio of 1 × 10^8^ CFU/g of feed, which is the mean of the dose range used for most of the probiotics (Newaj‐Fyzul & Austin, 2015). Treated shrimp were fed ad libitum three times a day during 30 days with the probiotic‐sprayed commercial diet, whereas the control group was fed with the commercial diet sprayed with sterile seawater (García‐Bernal, Medina‐Marrero, et al., 2017; García‐Bernal et al., 2018). The bacterial load in the food was confirmed by plate count; particulate matter was daily removed by siphon during the probiotic feeding period followed by the addition of the same amount of discarded water (25%), as reported in the preceding paper ( García‐Bernal, Medina‐Marrero, et al., 2017). No water exchange was performed during challenge, and dead animals were regularly removed from tanks throughout the daylight hours. Intestine samples for metagenomic studies were taken after the probiotic treatment (day 30) and at the end of *V. parahaemolyticus* CAIM 170 challenge (day 35, 5 days postchallenge) (Figure 1). Samples from probiotic‐fed shrimps were taken from the same amount of surviving animals as in the control (30% survival).

### 2.2 DNA extraction and sequencing

The DNA was extracted using the method of Sambrook, Fritsch, and Maniatis (1989). The complete intestinal tissue was homogenized in a lysis buffer containing Tris‐EDTA‐sodium dodecyl sulfate (SDS) (100 mmol/L); NaCl, 50 mmol/L; Tris (pH 8), 100 mmol/L; EDTA (pH 8); SDS (1%); and 100 µl of lysozyme (50 mg/ml; Sigma) at 37°C for one hour. Once homogenized, the tissue was incubated overnight at 65ºC with 20 µl of Proteinase K (20 mg/ml; Sigma), followed by the addition of 200 µl of 6 mol/L of NaCl, incubation on ice (20 min) and centrifugation (13,000 g, 4ºC, 10 min). The DNA was precipitated from the supernatant with absolute ethanol, left to settle overnight at 4°C and collected by centrifugation (8,000 g, 4ºC, 5 min). Extracted DNA was washed with 70% ethanol, dried, and resuspended in 50 μl of molecular grade water. DNA purity and concentration was determined with a NanoDrop 2000 spectrophotometer (Thermo Fisher Scientific™). Finally, DNA samples were stored until sequencing in the Laboratory for Microbial Genomics (Centro de Investigación en Alimentación y Desarrollo). To determine the microbiota present in the samples, the 16S rDNA variable region V3 was amplified by PCR (338F and 533R with Illumina adaptors) and barcoded following the protocol recommended by Illumina. Three 16S‐amplification replicas were performed to each DNA sample. Samples were quantified through Qubit Fluorometer and mixed together as an equimolar pool before sequencing in an Illumina Miniseq machine using standard conditions (300 cycles, 2 × 150).

### 2.3 Statistical analysis

Sequencing reads of the 16S rRNA gene were processed with QIIME software (Caporaso et al., 2010). Read preparation was performed with the pair‐end_cleaner v0.9.7 (https://github.com/GenomicaMicrob/pair-end_cleaner) program. The minimum sequence length was 170 bp, and singletons were discarded. Chimeric sequences were detected and eliminated with the program chimera_detector version 1.3.3 (https://github.com/GenomicaMicrob/). Metagenomic analysis was performed with the Microbiomal Helper (Comeau, Douglas, & Langille, 2017) program, using QIIME1 (Caporaso et al., 2010). The free chimera sequences were grouped in operational taxonomic units (OTUs) (97% identity). To assign OTUs, the script “pick_open_reference_otus.py” was used. The taxonomic data for each OTU were obtained from the reference bases using the script “assign_taxonomy.py.” Low confidence (0.1%) OTUs were removed with the script “remove_low_confidence_otus.py.” Rarefaction was performed with the script “single_rarefaction.py” utilizing the read count obtained as the lowest limit. Postrarefaction data allowed to calculate the relative abundance of the IM composition.

Alpha diversity was calculated through richness (Chao‐1) estimations as well as Shannon and Simpson indexes, using the script “alfa_diversity.py.” Comparisons among estimations were calculated with the software Past (Hammer, Harper, & Ryan, 2001). Diversity among groups (beta diversity) was estimated with weighted UniFrac implemented in the “beta_diversity.py” script and visualized graphically in a principal component analysis (PCA) plotted with EMPeror (Vazquez‐Baeza, Pirrung, Gonzalez, & Knight, 2013). Significant differences of the beta diversity estimates among and within groups were assessed with the nonparametric tests ANOSIM and PERMANOVA, using the average rank dissimilarity and the sum of squares of the distances between diversities, respectively, as implemented in the script “compare_categories.py” with 999 permutations and *p* < .05. The statistical differences of beta diversity were observed and plotted with the Statistical Analysis of Metagenomic Profiles (STAMP) (Parks, Tyson, Hugenholtz, & Beiko, 2014), using the Welch's test with correction of Benjamin Honchberg FDR (*q* value < 0.05).

## 3 RESULTS

### 3.1 Obtained sequences

To determine the bacterial microbiota composition of the gastrointestinal tract of shrimp fed with different *Streptomyces*‐based probiotics, a total of 25,000 valid sequences with an average read length of 170 bp were obtained by sequencing the V3 region of the 16S rRNA gene using Illumina platform. In general, bacterial OTUs from these sequences were assigned to 14 phyla, 49 families and 46 genera.

### 3.2 Richness and diversity analysis

Bacterial richness and diversity were estimated by Simpson, Shannon, and Chao‐1 indexes. Bacterial diversity in the groups RL8_ACH and Bac‐Strep_ACH (after challenge) with Shannon indexes of 3.94 ± 0.11 and 3.39 ± 0.3, respectively, was greater than the control groups. Chao‐1 values were higher in the groups RL8_ACH (368.5 ± 8.9) and Bac‐Strep_ACH (338.2 ± 19.2) than the Control_ACH (326.6 ± 15.8). Similarly, the groups RL8_ACH and Bac‐Strep_ACH with Simpson indexes of 0.95 ± 0.01 and 0.90 ± 0.02, respectively, exhibited higher diversity than the Control_ACH after *V. parahaemolyticus* challenge (0.78 ± 0.04) (Table 1).

**Table 1:** Diversity index (Shannon and Simpson) and OTU estimated richness (Chao1) for the intestinal microbiota of *Litopenaeus vannamei* before and after challenge with *Vibrio parahaemolyticus* and exposed to different probiotics

Treatments	OTU Number	Simpson_1‐D	Shannon_H	Chao−1
RL8_BCH	246	0.88 ± 0.03	3.06 ± 0.28	277.8 ± 10.9
RL8_ACH	330	0.95 ± 0.01	3.94 ± 0.11	368.5 ± 8.9
Lac‐Strep _BCH	220	0.85 ± 0.07	2.79 ± 0.22	295.3 ± 41.1
Lact‐Strep_ACH	238	0.81 ± 0.05	2.64 ± 0.28	283.4 ± 49.3
Bac‐Strep_BCH	205	0.78 ± 0.01	2.29 ± 0.09	259.4 ± 4.08
Bac‐Strep_ACH	296	0.90 ± 0.02	3.39 ± 0.3	338.2 ± 19.2
Control_BCH	193	0.88 ± 0.01	2.9 ± 0.03b	222 ± 65.05
Control_ACH	273	0.78 ± 0.04	2.58 ± 0.26	326.6 ± 15.8

Note

Data are expressed as media ± standard deviation.

Abbreviations: ACH, After challenge; BCH, Before challenge.

**Figure A1:**
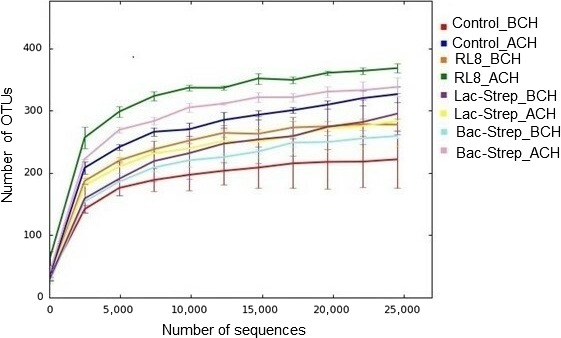
Rarefaction curves of bacterial richness in the intestines of *Litopenaeus vannamei* treated with *Streptomyces* alone [RL8] and combined with *Bacillus* [Bac‐Strep] and *Lactobacillus* [Lac‐Strep] before [ˍBCH] and after [ˍACH] challenge with *Vibrio parahaemolyticus*. Operational taxonomic units (OTUs) were defined as having 97% sequence identity as a function of the number of sequences analyzed

The rarefaction curve of the experimental groups reached the saturation plateau (Appendix Figure A1), which indicated that sampling captured the most representative bacterial richness present in shrimp intestine. In general, microbiota diversity in the majority of the experimental groups was higher after than before challenge with *V. parahaemolyticus*.

**Figure 2:**
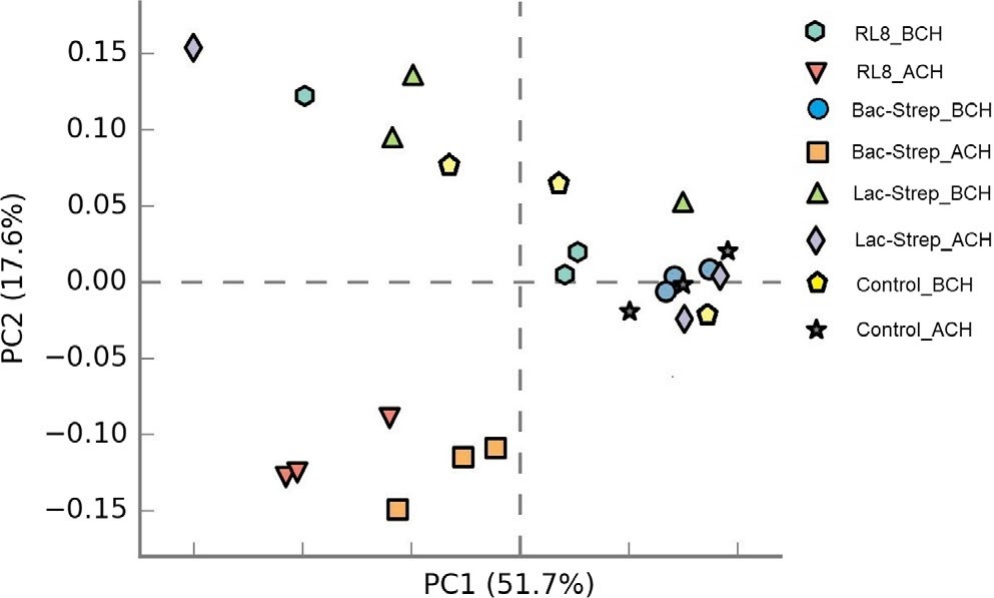
Principal components analysis (PCA) plot based on weighted UniFrac distance measurements of beta diversity associated with the gut microbiota of *Litopenaeus vannaemi* treated during 30 days with *Streptomyces* alone [RL8] and combined with *Bacillus* [Bac‐Strep] and *Lactobacillus* [Lac‐Strep]; before [ˍBCH] and after [ˍACH] challenge with *Vibrio parahaemolyticus*

Figure 2 shows the PCA of beta diversity associated with microbiota variance for groups treated with probiotics and control groups, before and after *V. parahaemolyticus* challenge. The principal components represented an accumulated variance of 69.3% (PC1 51.7% and PC2 17.6%). The results showed that after challenge, microbiota composition of shrimp treated with RL8 and Bac‐Strep was different from the rest of the experimental groups. These treatments were grouped on the left side of the chart along the first principal component axis (PC1). In contrast, shrimp IM from the rest of the groups showed a wider dispersion, indicating differences in beta diversity.

### 3.3 Composition of bacterial microbiota

**Figure 3:**
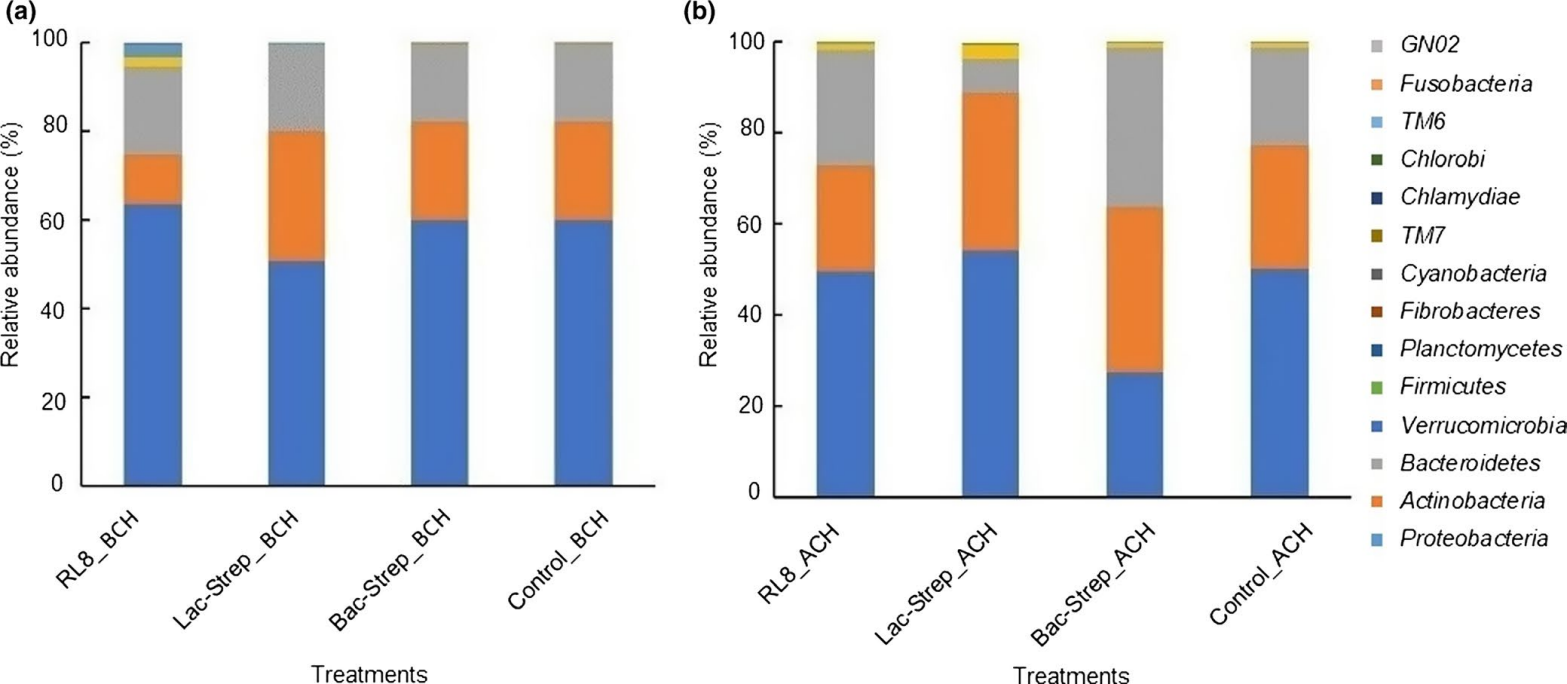
Relative abundance of different bacterial phyla associated with the gut microbiota of *Litopenaeus vannamei* treated during 30 days with Streptomyces alone [RL8] and combined with *Bacillus* [Bac‐Strep] and *Lactobacillus* [Lac‐Strep]; (a) before [ˍBCH] and (b) after [ˍACH] challenge with *Vibrio parahaemolyticus*. Relative abundance: percentage of each phylum with respect to all valid sequences for each treatment

The composition and abundance of the bacterial community of different experimental groups is shown in Figure 3. A total of 14 phyla were identified in the intestine of *L. vannamei*: *Proteobacteria*, *Actinobacteria*, *Bacteroidetes*, *Verrucomicrobia*, *Firmicutes*, *Planctomycetes*, *Fibrobacteres*, *Cyanobacteria*, *TM7*, *Chlamydiae*, *TM6*, *Chlorobi*, *Fusobacteria,* and *GNO2*. Except for Bac‐Strep_ACH, *Proteobacteria* was the most abundant bacterial phylum in all experimental groups before and after challenge (Figure 3a,b) with an average relative abundance of 45.34 ± 6.0% and 58.62 ± 2.74%, respectively. This phylum was followed by *Actinobacteria* and *Bacteroidetes* with relative abundances of 30.40 ± 3.11% and 21.21 ± 3.70% and, 22.15 ± 5.66% and 18.44 ± 0.73% before and after challenge, respectively (Figure 3a,b).

**Figure 4:**
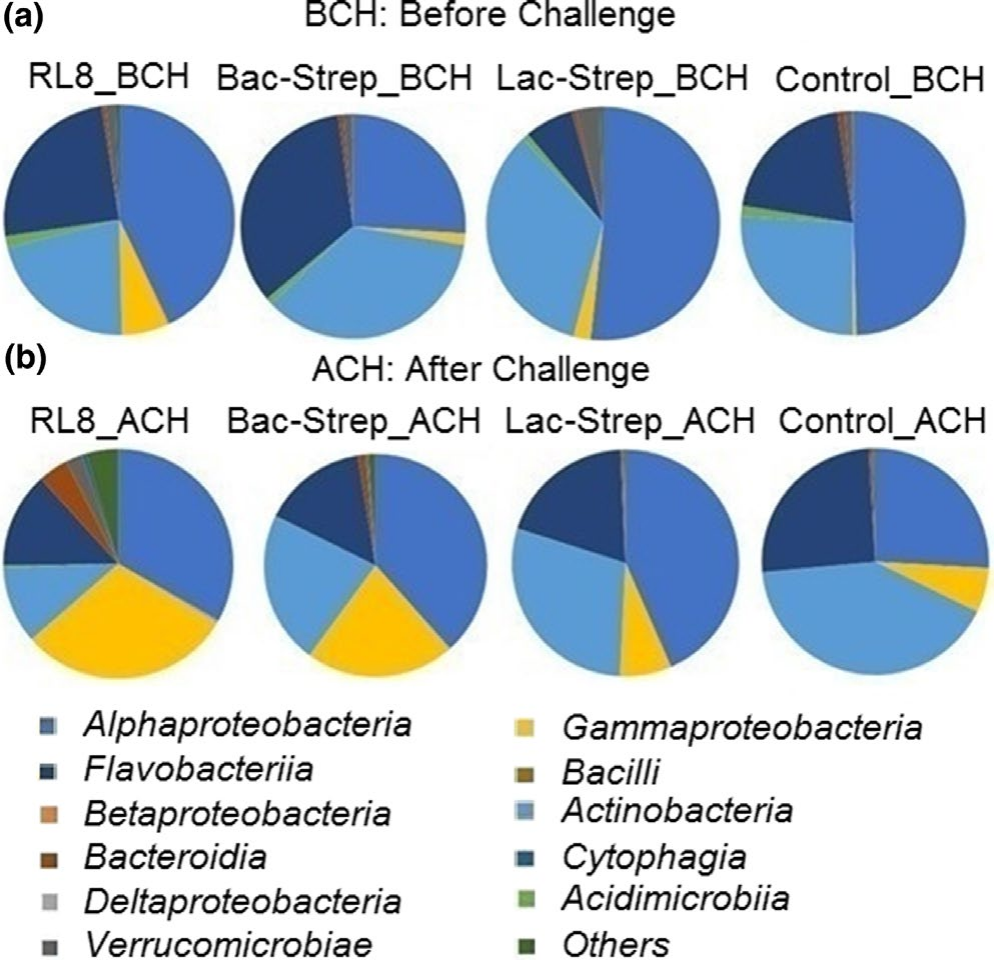
Relative abundance at class level associated with the gut microbiota of *Litopenaeus vannamei* treated during 30 days with *Streptomyces* alone [RL8] and combined with *Bacillus* [Bac‐Strep] and *Lactobacillus* [Lac‐Strep]; (a) before [ˍBCH] and (b) after [ˍACH] challenge with *Vibrio parahaemolyticus*. Relative abundance: percentage of each class compared with the total

The three bacterial classes with the highest relative abundance were *Alphaproteobacteria* with 42.5 ± 5.82%; *Actinobacteria* with 29.16 ± 3.30%; and *Flavobacteriia* with 21.45 ± 5.65% before challenge (BCH), and 35.23 ± 3.74%, 25.81 ± 6.35%, and 18.37 ± 0.92% after challenge (ACH), respectively (Figure 4a,b).

## 4 CHANGES IN BACTERIAL COMPOSITION

**Figure 5:**
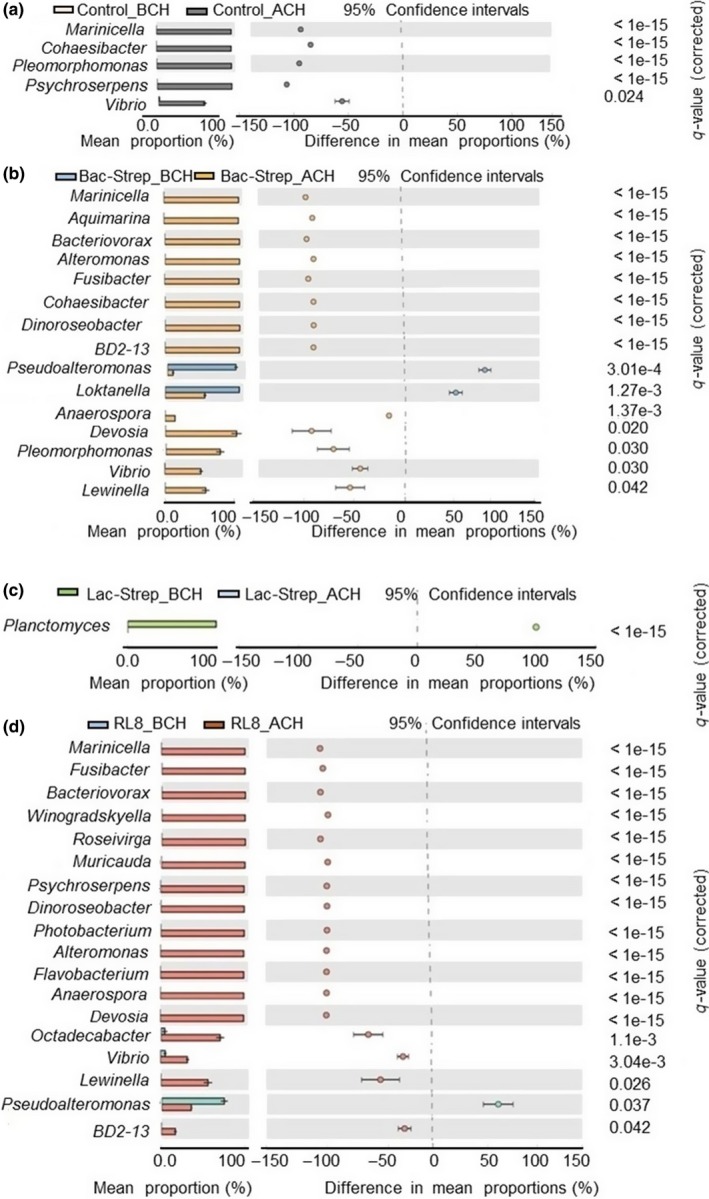
Comparative taxonomic profile at genus level of *Litopenaeus vannamei* juveniles untreated with probiotics (a) and treated during 30 days with Bac‐Strep (b), Lact‐Strep (c) and RL8 (d); before [ˍBCH] and after [ˍACH] *Vibrio parahaemolyticus* challenge. Analysis was performed with STAMP. The q‐values are based on Welsh's *t* test with correction of Benjamin Hochberg FDR (q value < 0.05)

ANOSIM and PERMANOVA analyses of shrimp microbiota showed significant differences (*p* = .0002 and *p* = .009, respectively), suggesting intra‐ and extra‐group variations at taxa level due to the effect of treatments. Those significant changes in the microbial communities were observed through the STAMP analysis, which showed that previous to Vibrio challenge only a few genera were dominant (Figure 5), including the genus *Planctomyces* in the Lac‐Strep group, *Pseudoalteromonas* in the RL8, and *Pseudoalteromonas* and *Loktanella* in the Bac‐Strep group. The diversity of predominant genera was much higher after Vibrio challenge even though no genus prevailed in the Lac‐Strep group. Among the five dominant genera present in the control group after challenge, *Marinicella* and *Vibrio* were also prevalent in the groups Bac‐Strep and RL8, *Cohaesibacter* and *Pleomorphomonas* in Bac‐Strep, and *Psychroserpens* in RL8. After challenge, *Bacteriovorax*, *Alteromonas*, *Fusibacter*, *Dinoroseobacter*, BD2‐13, *Anaerospora*, *Devosia,* and *Lewinella* constituted the core microbiota stimulated only by the probiotics Bac‐Strep and RL8. In general, 13 genera were stimulated in the Bac‐Strep group that also included the genus *Aquimarina*, and 17 in the RL8 group that included the genera *Winogradskyella*, *Roseivirga*, *Muricauda*, *Photobacterium*, *Flavobacterium,* and *Octadecabacter* (Figure 5).

## 5 DISCUSSION

The animals’ intestine is a vital organ for food storage, nutrient digestion, and absorption besides playing an important role in immunity (Ringø et al., 2016; Tzuc et al., 2014). Several intestinal functions are achieved through bacterial metabolism, which may also benefit the host by improving the immune response, nutrient absorption, and homeostasis maintenance (Hooper & Macpherson, 2010). Consequently, modulation of the IM, through optimization of diet formulation or supplementation with pre‐ and probiotics, is important to improve the general physiological development and increase the productivity and economic revenues during shrimp farming.

Bacterial diversity was estimated with the Shannon index whose highest values indicated a greater bacterial diversity (Luis‐Villasenor et al., 2013; Wang, Garrity, Tiedje, & Cole, 2007). The Shannon index values in this study showed that the groups RL8_ACH and Bac‐Strep_ACH (Shannon index of 3.94 and 3.39, respectively) had higher bacterial diversity, which has been associated with a greater host resistance to pathogen colonization (De Schryver & Vadstein, 2014) than the other experimental groups. Even though a significant change in microbiota composition of *L. vannamei* has also been achieved with other probiotics (Vargas‐Albores et al., 2017), this is the first study showing such effect with *Streptomyces* strains, either alone or combined. In contrast, the Shannon index in the Control_ACH group was 2.58 ± 0.26 after challenge with *V. parahaemolyticus*. This result suggested less bacterial diversity and species richness due to the presence of the pathogen and, thus, a greater susceptibility to invasion by this agent (Dillon, Vennard, Buckling, & Charnley, 2005). The results of this study differ from those obtained by Luis‐Villaseñor et al., (2013) who found a significant reduction of shrimp intestinal microbiota using a *Bacillus* mixture which suggested that the probiotic effect of *Bacillus* was strongly potentiated by the addition of *Streptomyces* strains.

*Proteobacteria* was the dominant phylum in the intestine of *L. vannamei* treated with probiotics before and after *V. parahaemolyticus* challenge, followed by *Actinobacteria* and *Bacteroidetes*. This phylum has been regarded as the most abundant in *L. vannamei* in multiple studies with relative abundances from 68% to 97% (Rungrassamee, Klanchui, Maibunkaew, & Karoonuthaisiri, 2016; Zheng et al., 2017). Similar results were also reported at different salinities (Zhang et al., 2016) and food types (Qiao et al., 2017). Other studies have detected the phyla *Firmicutes*, *Bacteroidetes,* and *Actinobacteria* as the most dominant after *Proteobacteria*. Nonetheless, the relative abundance of these bacteria in the intestine of *L. vannamei* changes according to the environment conditions and diet composition (Qiao et al., 2017; Tzuc et al., 2014; Zhang et al., 2014, 2016).

In this research, *Actinobacteria* was the second most abundant phylum in shrimp intestine. Some members of this phylum are known to be excellent secondary metabolite producers that can protect the host from an infection (Chau, Thanh, & Anh, 2016; Mahajan & Balachandran, 2012). Adding *Streptomyces* strains to feed resulted in a greater survival of *L. vannamei* after *V. parahaemolyticus* challenge (García‐Bernal, Medina‐Marrero, et al., 2017; García‐Bernal et al., 2018). These results, along with those of other authors, confirmed the great potential of *Streptomyces* strains as probiotic agents in aquaculture (Chau et al., 2016; Das, Ward, & Burke, 2008; García‐Bernal et al., 2015, 2018; García‐Bernal, Medina‐Marrero, et al., 2017; Kamarudheen, George, Pathak, George, & Rao, 2015; Tan, Chan, Lee, & Goh, 2016; Velmurugan et al., 2015).

*Alphaproteobacteria* was the most abundant class associated with *L*. *vannamei* before and after applying probiotics, followed by *Actinobacteria* and *Flavobacteriia*. Luis‐Villaseñor et al. (2015) investigated the effect of a mix of *Bacillus* and a commercial probiotic on *L. vannamei* IM; they demonstrated that the bacterial community of shrimps treated with probiotics consisted mainly of *Alpha* and *Gammaproteobacteria*, *Fusobacteria*, *Sphingobacteria,* and *Flavobacteriia*, whereas in the nontreated control group, *Alphaproteobacteria* and *Flavobacteriia* prevailed. Thus, these authors demonstrated that the IM composition of shrimps treated with the *Bacillus* mix was different from that of the control group. However, this was not the case in this study where both treated and control groups had similar dominant classes (Figure 4a,b).

One of the most significant results that derived from this study was the detection of members of the family *Bacteriovoracaceae*, mostly of the genus *Bacteriovorax* from the order *Bdellovibrionales* in the groups treated with RL8 and Bac‐Strep after *V. parahaemolyticus* challenge. *Bacteriovorax* is a small mobile predator bacteria that invades the periplasmic space of certain gram‐negative bacteria, including *Vibrio* species, altering the cellular wall of its prey, consuming the cytoplasmic content, and lysing the cell until it releases the predator progeny (Chen, Young, Berhane, & Williams, 2012; Crossman et al., 2013). These predator bacteria are often isolated from estuarine seawater (Pineiro et al., 2013) where they can attack and lyse a great variety of gram‐negative bacteria (Chen et al., 2012). As a consequence of its capacity to limit the proliferation of bacterial pathogens in aquatic systems (Li, Liu, Chen, Zhang, & Cai, 2011; Qi, Zhang, Boon, & Bossier, 2009), the order *Bdellovibrionales* is classified as one of the markers of good health status in shrimp larvae (Zheng et al., 2017). The results of our study are supported by recent studies, which showed that *Streptomyces* spp. RL8 and N7 stimulated the proliferation and maintenance of *Bacteriovorax* population in the oyster *Crassostrea sikamea* (García‐Bernal, Trabal‐Fernández, Saucedo‐Lastra, Medina‐Marrero, & Mazón‐Suástegui, 2017). Consequently, these *Streptomyces* strains could reduce pathogenic microorganisms in shrimp farms by virtue of their direct probiotic activity, as well as the indirect stimulatory effect on the predatory *Bacteriovorax* population, which should be corroborated under field conditions.

Apart from *Bacteriovorax*, the groups treated with RL8 and Bac‐Strep also stimulated a great diversity of bacterial genera after challenge, which included several antimicrobial secondary metabolite producers, such as *Planctomyces*, *Dinoroseobacter*, *Pseudoalteromonas,* and *Loktanella*, as well as some that showed quorum quenching properties, such as *Muricauda* (Figure 5) (Bentzon‐Tilia & Gram, 2017; Graca, Calisto, & Lage, 2016; Offret et al., 2016; Ranson et al., 2018; Y. Wang, Li, Cui, & Zhang, 2017). Even though the *Vibrio* genus was also detected in these groups, the stimulation of *Bacteriovorax* and of a great diversity of antimicrobial producers has resulted in an excellent protection against *V. parahaemolyticus* infection (García‐Bernal, Medina‐Marrero, et al., 2017). Indeed, *Streptomyces* sp. RL8 and its combination with bacilli (Bac‐Strep), as single‐ and multi‐strain probiotics, were capable of improving growth, immunological, and microbiological parameters (*Vibrio* count in water and hepatopancreas), as well as survival of *L. vannamei* under laboratory conditions (García‐Bernal, Medina‐Marrero, et al., 2017).

The Lac‐Strep group was the only *Streptomyces*‐containing group that did not stimulate *L*. *vannamei* microbiota after challenge with *V*. *parahaemolyticus*. This was unexpected since Lactobacilli are well‐known probiotics that may also modulate the shrimp microbiome (Li et al., 2018). The mechanism behind this nullifying effect is currently unknown. Thus, further interaction studies between *Streptomyces* spp. and *Lactobacillus* strains are required to determine the basis and role of such interactions on shrimp microbiome.

Although some *Vibrio* species, such as *V. harveyi* and *V. anguillarum*, are pathogens of penaeid shrimp and fish, respectively (Austin & Zhang, 2006; Frans et al., 2011), and others, such as *V. coralliilyticus* and *V. shiloi*, are coral pathogens, most species from this genus are benign (Thompson & Swings, 2006; Thompson & Polz, 2006). Therefore, it is likely that an important number of the *Vibrio* OTUs detected belonged to benign species, especially in those treated groups where an effective protection against *V. parahaemolyticus* was confirmed. Nevertheless, an important limiting factor of this study was the impossibility of identifying the different genera detected at species level, which prevented any comparison on the number of OTUs corresponding to *V. parahaemolyticus* among experimental groups.

The change in the microbiota composition of the control group with respect to probiotic‐fed groups cannot be attributed exclusively to *V*. *parahaemolyticus*, even though they were under the same experimental conditions after challenge with this agent. Instead, some indirect and stochastic effects which did not arise from the direct shrimp *V*. *parahaemolyticus* interaction may account for this variation (Zaneveld, McMinds, & Vega Thurber, 2017). By contrast, groups fed with the probiotics Rl8 and Bac‐Strept gained a more diverse and resilient microbial community composition that help them cope with the infection and any other detrimental stochastic effect.

Microbial colonization and survival in the intestines of targeted organisms are usually claimed as crucial prerequisites for potential probiotics (Lakshmi, Viswanath, & Sai Gopal, 2013). However, these conditions do not seem to be strictly required for shellfish organisms which can benefit from their continuous interaction with beneficial microorganisms thriving in the water and sediment (Li et al., 2018). This appears to be the case for *Streptomyces* sp. RL8 which is indigenous to the sea sediment, grows at a wide range of pH and salt concentrations, and produces resistant spores along with several extracellular enzymes and antimicrobial metabolites (García‐Bernal et al., 2015). Consequently, the modulatory effect on shrimp microbiome found here is not surprising, the same as several other probiotic effects already described for this strain (García‐Bernal, Medina‐Marrero, et al., 2017; García‐Bernal et al., 2018).

## 6 CONCLUSION

This study revealed that *Proteobacteria*, *Actinobacteria,* and *Bacteroidetes* were the predominant phyla in the intestine of the White shrimp *L. vannamei*. It also showed the modulating effect of *Streptomyces* sp. RL8 on *L. vannamei* microbiota, as well as its stimulatory effect on *Bacteriovorax* population and on several antimicrobial producers that protected shrimp from *V. parahaemolyticus* infection. This research contributed to a better understanding of the composition and dynamics of shrimp intestinal microbiota and the development of novel probiotics for the culture of this organism.

## CONFLICT OF INTERESTS

None declared.

## AUTHOR CONTRIBUTIONS

JMM‐S, MG‐B, and RM‐M conceived and designed the study. JMM‐S, MG‐B, and JS‐L performed the experiments. JS‐L carried out the bioinformatic analysis. MG‐B and RM‐G helped in the data analysis. MG‐B, RM‐M, JMM‐S, and RM‐G drafted the paper. All authors read and approved the final manuscript.

## ETHICS APPROVAL

The experiment complied with the Guidelines of the Mexican Government (NOM‐062—ZOO‐1999) for the production, care and use of experimental animals.

## Data Availability

All sequence data acquired during this investigation have been deposited in the NCBI Sequence Read Archive under project accession numbers MK588851‐MK589267.
